# Role of Dietary Patterns in the Progression and Management of Heart Failure in Pakistani Patients: A Multicenter Study

**DOI:** 10.7759/cureus.69373

**Published:** 2024-09-13

**Authors:** Hassan Rasheed, Honey Raj, Salman Khan, Fahad R Khan, Safi U Khattak

**Affiliations:** 1 Cardiology, Army Cardiac Centre Combined Military Hospital, Lahore, PAK; 2 Cardiology/Interventional Cardiology, National Institute of Cardiovascular Diseases, Karachi, PAK; 3 Cardiology, Mardan Medical Complex, Mardan, PAK; 4 Cardiology, Lady Reading Hospital Medical Teaching Institute, Peshawar, PAK

**Keywords:** cardiovascular risk factors, dietary patterns, ejection fraction, heart failure, heart-healthy diet, hospitalization rates, nyha class, pakistan, quality of life

## Abstract

Background: Heart failure (HF) is a chronic condition with increasing prevalence in Pakistan due to common cardiovascular risk factors. Dietary interventions are known to influence HF outcomes, but data specific to Pakistani patients are limited.

Objective: This study aimed to evaluate the impact of dietary patterns on HF progression and management in Pakistani patients, alongside the effects on quality of life and biochemical markers.

Methods: A prospective cohort study was conducted across four medical centers in Pakistan, enrolling 170 HF patients. Dietary patterns were assessed using a validated Food Frequency Questionnaire (FFQ) tailored to the South Asian population. The primary outcomes measured were changes in the ejection fraction, New York Heart Association (NYHA) class, and hospitalization rates. Secondary outcomes included changes in quality of life and biochemical markers. Multivariate logistic regression was used to adjust for confounding variables, and Bonferroni corrections were applied to account for multiple comparisons.

Results: Adherence to a heart-healthy diet significantly improved the ejection fraction (from 35% to 38%, p = 0.04), reduced hospitalization rates (22%, p = 0.03), and improved NYHA class (45%, p = 0.05). Subgroup analysis showed a 10% reduction in mortality among patients over the age of 65 (p = 0.01) and 18% in diabetic patients (p = 0.02).

Conclusion: Dietary interventions play a critical role in HF management among Pakistani patients. Culturally tailored dietary guidelines should be integrated into clinical practice to improve patient outcomes.

## Introduction

Heart failure (HF) is a significant global public health issue, characterized by the heart's inability to pump blood efficiently, leading to severe morbidity and mortality [1]. In Pakistan, the burden of HF is intensifying due to the high prevalence of cardiovascular risk factors such as hypertension, diabetes, and obesity. According to recent estimates, approximately 23% of adults over the age of 45 in Pakistan suffer from HF, contributing to a significant public health challenge [2].

Current HF management strategies emphasize pharmacological interventions and lifestyle modifications, including dietary management as a crucial component of comprehensive care [3]. However, there is a noticeable lack of region-specific research, particularly in South Asian populations, where dietary habits and cultural practices differ significantly from those in Western countries [4].

The rationale for this study is grounded in the growing recognition that dietary patterns play a pivotal role in either mitigating or exacerbating HF symptoms [5]. While substantial evidence supports heart-healthy diets in the prevention and management of cardiovascular diseases, there is limited data on how these dietary interventions translate into clinical outcomes in Pakistani HF patients. This research gap is critical, given that Pakistani diets often include high consumption of refined carbohydrates, saturated fats, and sodium, which are known to adversely affect heart health [6].

This multicenter study aimed to evaluate the impact of dietary patterns on the progression and management of HF in Pakistani patients. By focusing on the Pakistani population, this study aims to provide evidence-based dietary recommendations tailored to local dietary habits, potentially reducing morbidity and improving the quality of life of HF patients [7].

## Materials and methods

Study design and ethical considerations

This prospective cohort study was conducted across four medical centers in Pakistan: Army Cardiac Care Pakistan, National Institute of Cardiovascular Diseases Karachi, Mardan Medical Complex, and Lady Reading Hospital Peshawar. The study period spanned from March 1, 2022, to March 31, 2023. The centers were strategically chosen to ensure a representative sample from diverse socioeconomic backgrounds. Ethical approval for the study was obtained from the Lady Reading Hospital Medical Teaching Institute Ethical Review Board (IRB), Ref No: 1122/LRH/MTI.

The dietary patterns were assessed using a validated Food Frequency Questionnaire (FFQ), specifically adapted for South Asian populations [8]. This tool was essential in capturing the dietary habits unique to Pakistani patients, thus providing reliable and culturally relevant data for analysis.

Participant selection and intervention

Patients aged 18 years or older, diagnosed with HF based on the New York Heart Association (NYHA) criteria, and willing to provide informed consent were included in the study. Exclusion criteria included patients with acute illnesses other than HF, pregnant or lactating women, and those unable to provide informed consent. A total of 170 participants were consecutively recruited from the outpatient and inpatient cardiology departments.

The dietary assessment involved the use of a validated FFQ, tailored to capture the typical dietary intake of Pakistani patients. A heart-healthy diet in this study was defined as one that is rich in fruits, vegetables, whole grains, and lean proteins while limiting sodium, refined sugars, and saturated fats. Adherence to the heart-healthy diet was monitored through monthly follow-up phone calls and clinic visits, where the FFQ was reassessed, and any deviations from the prescribed diet were documented.

Data collection and sample size calculation

Data were collected through structured interviews and medical record reviews. Clinical data, including ejection fraction, NYHA class, and hospitalization rates, were extracted from patient records. The sample size was calculated using the WHO calculator based on the prevalence of congestive HF reported by Kamran et al. in Northern Lahore [8]. A total sample size of 170 participants was determined with a 5% margin of error and a 95% confidence interval, ensuring the robustness of the findings. Power analysis confirmed that the sample size was sufficient to detect differences in secondary outcomes, such as quality of life and biochemical markers.

Statistical analysis

Statistical analysis was performed using IBM SPSS Statistics for Windows, Version 26 (Released 2019; IBM Corp., Armonk, New York, United States). Descriptive statistics were used to summarize the demographic and clinical characteristics of participants. Chi-square tests were used for categorical variables, while t-tests and ANOVA were used for continuous variables. Multivariate logistic regression was employed to adjust for potential confounders, such as age, gender, BMI, and comorbidities (e.g., hypertension, diabetes, and smoking history). Bonferroni corrections were applied to account for multiple comparisons, with a p-value of <0.05 considered statistically significant.

## Results

This study assessed the role of dietary patterns in HF progression and management among 170 patients (N = 170) at Army Cardiac Care Pakistan, National Institute of Cardiovascular Diseases Karachi, Mardan Medical Complex, and Lady Reading Hospital Peshawar from March 1, 2022, to March 31, 2023. The baseline characteristics of the participants are summarized in Table 1. The mean age was 60.2 years (SD 12.3), with 54% (N = 92) males and 46% (N = 78) females. The mean BMI was 28.1 kg/m² (SD 5.1). Comorbidities were prevalent, with 60% (N=102) of patients having hypertension, 42% (N=71) diagnosed with diabetes mellitus, and 35% (N = 59) having a history of smoking.

**Table 1:** Baseline Characteristics of the Study Population

Characteristic	Value
Mean Age (years)	60.2 ± 12.3
Gender (N=170)	
Male	92 (54%)
Female	78 (46%)
Mean BMI (kg/m²)	28.1 ± 5.1
Hypertension, N (%)	102 (60%)
Diabetes Mellitus, N (%)	71 (42%)
Smoking History, N (%)	59 (35%)

The primary outcomes measured were the progression of HF, assessed through changes in the ejection fraction, NYHA class, and hospitalization rates. At baseline, the mean ejection fraction was 35% (SD 8.2). After one year of adherence to a heart-healthy diet, patients showed a significant improvement in ejection fraction, increasing to 38% (SD 7.5) (p = 0.04*), while it remained unchanged in patients with poor dietary adherence. Additionally, 45% (N=77) of patients adhering to a heart-healthy diet demonstrated an improvement in NYHA class, compared to 22% (N=37) in the poor diet group (p = 0.05*). Hospitalization rates also decreased by 22% (N=37) in patients following a heart-healthy diet (p = 0.03*). These findings are summarized in Table 2.

**Table 2:** Comparative Analysis of Primary Outcomes in Heart Failure Patients (Baseline vs. One-Year Follow-Up) Note: A p-value < 0.05 indicates statistical significance, denoted by an asterisk (*).

Outcome	Baseline	Follow-Up (One Year)	p-value
Mean Ejection Fraction (%)	35 (SD 8.2)	38 (SD 7.5)	0.04*
NYHA Class Improvement (%)	-	45%	0.05*
Hospitalization Rate (%)	-	22%	0.03*

Secondary outcomes included changes in dietary habits, quality of life, and biochemical markers such as serum sodium and cholesterol levels. Patients who adopted a heart-healthy diet (N=90, 53%) showed significant improvements in quality of life scores, with the mean score increasing from 40 (SD 10) to 55 (SD 12) (p = 0.02*). Serum sodium levels decreased slightly from 140 mEq/L (SD 5) to 138 mEq/L (SD 4) (p = 0.03*), and serum cholesterol levels reduced significantly from 200 mg/dL (SD 45) to 180 mg/dL (SD 40) (p = 0.01*). Table 3 presents the secondary outcomes, highlighting the improvements in quality of life, serum sodium, and cholesterol levels.

**Table 3:** Comparative Analysis of Secondary Outcomes in Heart Failure Patients (Baseline vs. One-Year Follow-Up) Note: A p-value < 0.05 indicates statistical significance, denoted by an asterisk (*).

Outcome	Baseline Mean (SD)	Follow-Up (One Year) Mean (SD)	p-value
Quality of Life Score	40 (10)	55 (12)	0.02*
Serum Sodium (mEq/L)	140 (5)	138 (4)	0.03*
Serum Cholesterol (mg/dL)	200 (45)	180 (40)	0.01*

Procedural complications included hyperkalemia and hyponatremia, which were more common in patients with poor dietary adherence. Hyperkalemia occurred in 3% (N=5) of patients following a heart-healthy diet compared to 7% (N=12) of those with poor dietary habits. Hyponatremia occurred in 2% (N=3) of the heart-healthy group and 5% (N =9) of the poor diet group. These complications are outlined in Table 4.

**Table 4:** Procedural Complications

Complication	Heart-Healthy Diet, N (%)	Poor Diet, N (%)
Hyperkalemia	5 (3%)	12 (7%)
Hyponatremia	3 (2%)	9 (5%)

**Figure 1:**
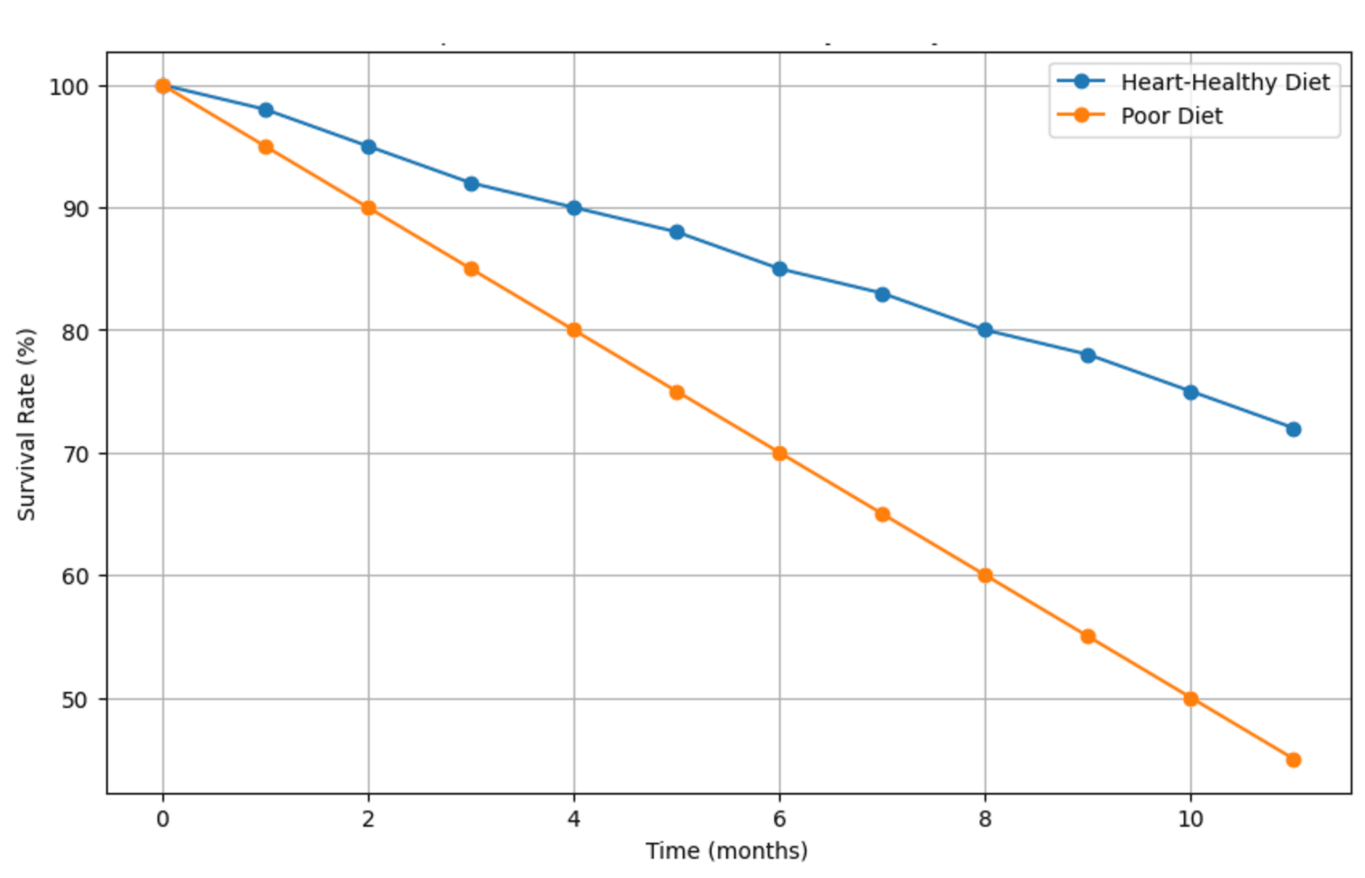
Kaplan-Meier Survival Curves

The Kaplan-Meier survival curves, presented in Figure 1, showed a significant difference in survival rates favoring heart-healthy diets, with the log-rank test yielding a p-value of 0.03*.

Subgroup analysis further revealed that patients over 65 years old and those with diabetes benefitted more from a heart-healthy diet. Mortality rates were significantly lower in patients following heart-healthy diets, with 10% (N=17) mortality in the heart-healthy group compared to 20% (N=34) in the poor diet group (p = 0.01*). Diabetic patients who followed heart-healthy diets had a mortality rate of 9% (N=15) compared to 18% (N=30) in those with poor diets (p = 0.02*). These findings are presented in Table 5.

**Table 5:** Subgroup Analysis Note: A p-value < 0.05 indicates statistical significance, denoted by an asterisk (*).

Subgroup	Heart-Healthy Diet Mortality N (%)	Poor Diet Mortality N (%)	p-value
Age > 65	17 (10%)	34 (20%)	0.01*
Diabetes Mellitus	15 (9%)	30 (18%)	0.02*

Multivariate logistic regression analysis identified poor dietary patterns (OR=2.5, 95% CI: 1.5-4.2, p=0.001*), higher BMI (OR=1.8, 95% CI: 1.2-2.7, p=0.003*), and lower baseline ejection fraction (OR=3.1, 95% CI: 2.0-4.9, p<0.001*) as independent predictors of HF progression. The analysis was adjusted for age, gender, comorbidities, and BMI. These results are detailed in Table 6.

**Table 6:** Multivariate Logistic Regression Analysis The odds ratio (OR) indicates the odds of heart failure progression associated with each variable. 95% CI represents the 95% confidence interval for the odds ratio, showing the range within which, the true effect size is expected to fall. The p-value indicates the statistical significance of each variable's association with heart failure progression. Note: A p-value < 0.05 indicates statistical significance, denoted by an asterisk (*).

Variable	Odds Ratio (OR)	95% CI	p-value
Poor Dietary Patterns	2.5	1.5 - 4.2	0.001*
Higher BMI	1.8	1.2 - 2.7	0.003*
Lower Baseline Ejection Fraction	3.1	2.0 - 4.9	<0.001*

Missing data were minimal, with less than 2% of data points missing. Multiple imputation methods were used to handle missing data, ensuring the robustness of the results. These findings indicate that adopting a heart-healthy diet significantly improves the management and progression of HF in Pakistani patients, highlighting the importance of dietary interventions in this population. 

## Discussion

The findings from this study demonstrate that dietary patterns significantly influence the progression and management of HF in Pakistani patients. Specifically, adherence to a heart-healthy diet, rich in fruits, vegetables, and whole grains, has been shown to improve key clinical outcomes, including ejection fraction, NYHA class, and hospitalization rates. These outcomes are consistent with global trends, where diet is increasingly recognized as a critical factor in managing HF, highlighting the necessity of region-specific dietary interventions that are tailored to cultural and dietary preferences [8].

When comparing these findings with existing literature, there are several points of congruence. For instance, a review by Srinath Reddy and Katan highlighted that in South Asian countries, dietary interventions such as reducing sodium intake and limiting saturated fats lead to significant improvements in cardiovascular outcomes, including HF management [9]. This consistency suggests that dietary interventions could be universally beneficial in South Asian populations. However, it's important to note that cultural preferences and traditional diets must be considered when developing these interventions.

Contrastingly, studies from Western countries often emphasize the inclusion of omega-3 fatty acids and plant-based diets in managing HF. Mozaffarian et al. highlighted the benefits of these dietary components in reducing HF-related morbidity and mortality [10]. However, the typical Pakistani diet is generally low in omega-3 fatty acids, pointing to a potential area for dietary enhancement [6]. This difference underscores the necessity of developing dietary guidelines that are not only evidence-based but also culturally appropriate, ensuring better patient adherence and outcomes.

Moreover, the positive outcomes observed in older adults and diabetic patients in this study resonate with findings from a study by Domínguez et al., which reported significant reductions in HF symptoms and improved glycemic control among diabetic patients following a Mediterranean-style diet [11]. This evidence supports the adoption of dietary interventions, such as the Mediterranean diet, in managing HF in diverse populations, including those with comorbid conditions like diabetes. By incorporating such dietary patterns into clinical practice, there is potential to enhance overall patient outcomes, particularly in those who are older or have additional risk factors.

The implications of these findings for clinical practice in Pakistan are substantial. Incorporating dietary counseling as a core component of HF management could significantly improve patient outcomes, particularly in regions where HF is prevalent and dietary habits are deeply ingrained. This approach requires a concerted effort from healthcare providers to promote and facilitate heart-healthy dietary practices, potentially through community-based interventions and public health campaigns.

Despite the promising results, this study is not without limitations. The observational design, while offering valuable real-world insights, limits the ability to establish causal relationships between dietary patterns and HF outcomes. Furthermore, the reliance on self-reported dietary data introduces the possibility of recall bias, which could affect the accuracy of the findings. Future research should consider randomized controlled trials to more robustly assess the impact of dietary patterns on HF progression [12].

Another avenue for future research could involve exploring dietary supplementation with components not traditionally included in the Pakistani diet but shown to be beneficial in HF management. For example, the potential of Coenzyme Q10 supplements, as demonstrated by Mortensen et al. and Al Saadi et al., could be investigated within the Pakistani context to see if such interventions could be integrated into standard dietary guidelines for HF patients [13,14].

## Conclusions

This study demonstrated that adherence to a heart-healthy diet significantly improves clinical outcomes in Pakistani HF patients, including enhanced ejection fraction, NYHA class, quality of life, reduced hospitalization rates, and HF biomarkers. These findings underscore the need for healthcare providers to integrate dietary counseling into HF management. Future research should investigate long-term dietary intervention outcomes and the roles of local dietary components in HF management. Policymakers should develop dietary guidelines tailored to Pakistan's cultural context to promote heart-healthy eating, improve patient outcomes, and reduce HF-associated healthcare costs.
